# Evaluating trends and outcomes between robotic and laparoscopic bariatric surgery in patients with BMI ≥ 60 kg/m^2^: an MBSAQIP analysis of 32,295 cases

**DOI:** 10.1007/s00464-026-12652-5

**Published:** 2026-03-12

**Authors:** Pattharasai Kachornvitaya, Mélissa V. Wills, Juan S. Barajas-Gamboa, Xinlei Zhu, Yung Lee, Suthep Udomsawaengsup, Salvador Navarrete, Ricard Corcelles, Andrew Strong, Matthew Kroh, Jerry Dang, Valentin Mocanu

**Affiliations:** 1 Digestive Disease and Surgery Institute, Cleveland Clinic, Cleveland, OH USA; 2 Cleveland Clinic Lerner College of Medicine of Case Western Reserve University, Cleveland, OH USA; 3 Digestive Diseases Institute, Cleveland Clinic Abu Dhabi, Abu Dhabi, United Arab Emirates; 4 Treatment of Obesity and Metabolic Disease Research Unit, Faculty of Medicine, Chulalongkorn University, Bangkok, Thailand; 5 Department of Surgery, Faculty of Medicine, Chulalongkorn University, Bangkok, Thailand

**Keywords:** BMI ≥ 60 kg/m2, Robotic, Sleeve gastrectomy, Roux-en-Y gastric bypass, Perioperative outcomes Risk factors

## Abstract

**Background:**

Bariatric surgery patients with body mass index (BMI) ≥ 60 kg/m2 present unique technical and perioperative challenges. While robotic-assisted bariatric surgery is thought to offer potential technical advantages, direct comparisons between robotic and laparoscopic approaches (R-BS and L-BS) in this population remains limited.

**Methods:**

An analysis of the 2020–2023 MBSAQIP database was conducted and all patients with BMI ≥ 60 kg/m2 who underwent primary laparoscopic or robotic sleeve gastrectomy (SG) and Roux-en-Y gastric bypass (RYGB) were included. Baseline demographics, operative characteristics, and 30-day postoperative outcomes were compared. Multivariable logistic regression identified independent predictors of serious complications.

**Results:**

Of 32,295 patients, 22,211 (68.8%) were L-BS and 10,084 (31.2%) were R-BS. Significant baseline differences existed between groups, including higher rates of gastroesophageal reflux disease (28.6% vs. 26.1%, p < 0.001), and hypertension (54.7% vs. 52.8%, p = 0.001) in the R-BS group. From 2020 to 2023, the proportion of R-BS doubled from 20.4% to 41.3%, whereas the proportion of L-BS declined slightly from 79.6% to 58.7%. There was no significant difference in robotic versus laparoscopic utilization for RYGB. (27.4% vs 26.4*%, *p = 0.059) and operative time was significantly longer in R-BS (106.5 ± 51.4 min vs. 83.5 ± 47.0 min, p < 0.001). Rates of individual 30-day complications, including leaks, bleeding, reoperation, and readmission, were low with no significant difference between cohorts. Independent predictors of serious complications included older age, hypertension, gastroesophageal reflux disease, prior myocardial infarction, therapeutic anticoagulation, longer operative time and RYGB. The robotic approach was neither independently associated with nor protective against serious complications.

**Conclusions:**

In patients with a BMI ≥ 60 kg/m2 undergoing elective bariatric surgery, there were no significant differences in 30-day postoperative outcomes between laparoscopic and robotic approaches despite baseline patient differences between groups. Although operative times were 27% longer for the robotic approach, its utilization increased substantially over the study period. These findings suggest that perioperative outcomes in this high-risk population are primarily determined by patient comorbidities and procedural factors rather than surgical approach, and that neither approach demonstrates superior short-term safety.

**Supplementary Information:**

The online version contains supplementary material available at 10.1007/s00464-026-12652-5.

The global prevalence of obesity continues to rise, with severe obesity, particularly among individuals with a body mass index (BMI) ≥ 60 kg/m2, emerging as a growing public health challenge. Metabolic and bariatric surgery, most commonly performed laparoscopically, offers significant benefits for these patients. which are most frequently performed with the laparoscopic approach. However, patients with BMI ≥ 60 kg/m2 present unique technical challenges, including limited intra-abdominal workspace, increased anesthetic risk, longer operative times, longer hospital lengths of stay, and elevated postoperative complication rates [1–3, 10]. Over the past two decades, the prevalence of individual in the United States with a BMI ≥ 60 kg/m2 has markedly increased, rising from 0.12% in 2001–2004 to 0.37% in 2021–2023 [4]. This population now comprising an increasing proportion of bariatric surgery candidates and often require specialized perioperative management and advanced surgical expertise. Laparoscopic bariatric surgery (L-BS), including sleeve gastrectomy (SG) and Roux-en-Y gastric bypass (RYGB), can be technically demanding due to increased visceral adiposity, limited visualization, and restricted instrument maneuverability [5].

Robotic-assisted bariatric surgery (R-BS) has emerged as a promising alternative, offering improved ergonomics, enhanced dexterity, and better visualization, which may be particularly advantageous in patients with BMI ≥ 60 kg/m2 [1, 6]. However, whether these technical advantages translate into superior perioperative outcomes remains uncertain in this specific high-risk population. National data show a rapid increase in robotic bariatric procedures across all BMI categories, with one MBSAQIP study reporting a rise in robotic utilization from 6% to 17.2% between 2015 and 2020 [7]. Similar increases have been observed in revisional and technically complex cases, suggesting that robotic systems are being increasingly utilized in both routine and more technically challenging bariatric surgery scenarios [8, 9]. However, the adoption patterns and outcomes specifically in this cohort remain poorly characterized.

Existing comparative studies report mixed findings: some show no significant differences in morbidity, mortality, or complications between laparoscopic and robotic approaches [10–13], while others suggest potential advantages of robotic RYGB, including reduced anastomotic leak rates in high-BMI patients [14]. Concerns about operative time and costs persist, though recent data suggest these differences may narrow with experience, particularly in super-obese cohorts [15]. Importantly, most studies have included few patients with BMI ≥ 60 kg/m2, limiting applicability to this highest-risk group. This study aims to address this gap by analyzing MBSAQIP data to compare perioperative outcomes of robotic versus laparoscopic bariatric surgery in this group. The primary objective was to assess whether robotic assistance confers superior short-term safety outcomes; secondary objectives were to evaluate national utilization trends and identify predictors of adverse postoperative outcomes.

## Materials and methods

### Data source and ethical approvals

A retrospective analysis was conducted using the MBSAQIP Participant Use File (2020–2023), which includes prospectively collected data from nearly 1,000 accredited bariatric centers in the U.S. and Canada. The dataset captures standardized pre-, intra-, and postoperative variables for bariatric surgery. Institutional Review Board approval was not required due to the de-identified nature of the dataset.

### Patient variables and population

Adult patients with a BMI ≥ 60 kg/m2 who underwent primary laparoscopic or robotic SG or RYGB were included. SG was identified using CPT code 43775; RYGB was identified using CPT code 43644; All revisional, conversion, and multi-stage procedures were excluded to focus specifically on primary bariatric operations. Patients with incomplete demographic data, missing procedural information, or indeterminate surgical approach were excluded from analysis.

Demographic data (age, sex, race, BMI, smoking status, functional status, ASA class), comorbidities were extracted and categorized by system: metabolic (e.g., diabetes, hypertension), cardiovascular, pulmonary, renal, and others (e.g., GERD, VTE history, anticoagulant use), and perioperative details: operative time (skin incision to skin closure) and length of stay. All variables followed MBSAQIP definitions. The number of operations performed in each operative year was also reported for both groups.

### Objectives

The primary objective was to evaluate the safety profile and complication rates of R-BS compared to L-BS using a composite of serious 30-day complications, including anastomotic leak, bleeding, reoperation, non-operative intervention, cardiac events, cerebrovascular accident, pneumonia, bowel obstruction, unplanned intubation, readmission, acute renal failure, VTE, deep surgical site infection, wound disruption, sepsis, or death. This composite outcome was designed to capture clinically significant morbidity that would impact patient recovery and healthcare resource utilization, consistent with previous MBSAQIP analyses. Individual components were also analyzed separately to identify specific areas of difference.

Secondary objectives were to evaluate temporal trends for both approaches and to determine independent predictors of serious complications.

### Statistical analysis

Categorical variables were summarized as percentages, and continuous variables as means ± standard deviation. Group differences were tested using chi-square and independent t-tests. Univariate analyses were performed for the combined cohort as well as for SG and RYGB subgroups. Multivariable logistic regression identified predictors of serious complications and mortality, were conducted for the combined cohort only. Variables with p < 0.05 in univariate analysis were included in multivariable models. Multicollinearity was assessed using variance inflation factors (VIF > 5).

Model performance was evaluated using area under the receiver operating characteristic (AUROC) curve (acceptable if > 0.6) and Brier score (< 0.1 indicates good calibration). A two-sided p-value < 0.05 was considered statistically significant. Missing data (< 5% for all variables) were addressed using available case analysis. All analyses were performed using STATA 17 (StataCorp, College Station, TX).

## Results

### Patient baseline characteristics

A total of 32,295 patients with BMI > 60 kg/m2 were included: 22,211 (68.8%) underwent L-BS and 10,084 (31.2%) underwent R-BS (Table 1). The mean age was slightly higher in the R-BS group (39.8 ± 10.6 vs. 39.6 ± 10.7 years; p = 0.041), and a greater proportion were female (74.6% vs. 73.4%; p= 0.020). R-BS utilization increased by 20.9 percentage points over the study period, from 20.4% in 2020 to 41.3% in 2023, while the proportion of L-BS decreased by a corresponding 20.9 percentage points, from 79.6% to 58.7%. (Table 2). Baseline comorbidities were comparable between groups, although R-BS patients had higher rates of gastroesophageal reflux disease (28.6% vs. 26.1%; p < 0.0001), hypertension (54.7% vs. 52.8%; p = 0.001), and chronic obstructive pulmonary disease (2.3% vs. 1.8%; p = 0.004). R-BS patients also had higher rates of hyperlipidemia (18.8% vs. 17.8%, p = 0.033) and obstructive sleep apnea (55.5% vs. 54.4%, p = 0.049). Despite these differences, the absolute differences were small, with most comorbidities differing by less than 2 percentage points between groups.

**Table 1:** Baseline characteristics of the patients

	Laparoscopic BS (n = 22,211)	Robotic BS (n = 10,084)	p-value
Age (years, mean ± SD)	39.6 ± 10.7	39.8 ± 10.6	0.041
Female	16,298 (73.4)	7,523 (74.6)	0.020
Race			< 0.0001
White	12,861 (57.9)	5,593 (55.5)	
Black or African American	6,624 (29.8)	3,258 (32.3)	
Other	2,726 (12.3)	1,233 (12.2)	
Body mass index (kg/m2, mean ± SD)	65.9 ± 6.0	66.2 ± 6.1	0.001
Functional status			0.819
Independent	21,733 (98.1)	9,869 (98.0)	
Partially dependent	400 (1.8)	183 (1.8)	
Dependent	27 (0.1)	15 (0.2)	
ASA category			< 0.0001
ASA 1–2	1,127 (5.1)	415 (4.1)	
ASA 3	18,030 (81.3)	8,059 (80.0)	
ASA 4–5	3,032 (13.6)	1,602 (15.9)	
Smoker in the past year	1,734 (7.8)	728 (7.2)	0.065
Diabetes			0.016
None or diet controlled	16,258 (73.2)	7,228 (71.7)	
Non-insulin dependent	4,531 (20.4)	2,185 (21.7)	
Insulin dependent	1,422 (6.4)	671 (6.7)	
Hypertension	11,719 (52.8)	5,517 (54.7)	0.001
Gastroesophageal reflux disease	5,789 (26.1)	2,879 (28.6)	< 0.0001
Hyperlipidemia	3,961 (17.8)	1,898 (18.8)	0.033
Obstructive sleep apnea	12,071 (54.4)	5,599 (55.5)	0.049
Chronic obstructive pulmonary disease	408 (1.8)	234 (2.3)	0.004
Immunosuppressive therapy	461 (2.1)	219 (2.2)	0.577
Renal insufficiency	120 (0.5)	48 (0.5)	0.457
History of venous thromboembolism	1,063 (4.8)	486 (4.8)	0.896
Venous stasis	504 (2.3)	207 (2.1)	0.219
Therapeutic anticoagulation use	1,181 (5.3)	533 (5.3)	0.907
History of myocardial infarction	177 (0.8)	87 (0.9)	0.464
Previous PCI	199 (0.9)	90 (0.9)	0.976
Previous major cardiac surgery	129 (0.6)	44 (0.4)	0.099

*BS* bariatric surgery, *ASA* American Society of Anesthesiologists, *PCI* percutaneous coronary intervention

**Table 2:** Annual distribution of laparoscopic and robotic BS in patients with BMI > 60 kg/m2 from 2020 to 2023

Operative year	Laparoscopic BS	Robotic BS	Total
2020	5,284 (79.6)	1,358 (20.4)	6,642
2021	6,002 (73.9)	2,118 (26.1)	8,120
2022	5,863 (65.8)	3,045 (34.2)	8,908
2023	5,062 (58.7)	3,563 (41.3)	8,625

*BS* bariatric surgery

### Procedural details and postoperative complications

RYGB was performed in 27.4% of R-BS and 26.4% of L-BS cases (p = 0.059) (Table 3). Operative time was significantly longer for R-BS (106.5 ± 51.4 min) compared to L-BS (83.5 ± 47.0 min; p < 0.001). Length of stay was slightly shorter in the R-BS group (1.4 ± 1.4 vs. 1.5 ± 1.5 days; p = 0.0013). Operative time trends by procedure and platform (2020–2023) are shown in Supplementary Fig. 1.

**Table 3:** Perioperative details and 30-day postoperative complications

	Laparoscopic BS (n = 22,211)	Robotic BS (n = 10,084)	p-value
Operative details			
RYGB, *n* (%)	5,872 (26.4)	2,767 (27.4)	0.059
Operative time (mins), mean ± SD	83.5 ± 47.0	106.5 ± 51.4	< 0.0001
Length of stay (days), mean ± SD	1.5 ± 1.5	1.4 ± 1.4	0.0013
Surgical complications, *n* (%)			
Readmission	739 (3.3)	373 (3.7)	0.090
Reoperation	186 (0.8)	87 (0.9)	0.818
Non-operative intervention	155 (0.7)	76 (0.8)	0.581
Leaks	58 (0.3)	19 (0.2)	0.214
Postoperative bleeding	213 (1.0)	106 (1.1)	0.438
Bowel obstruction	31 (0.1)	18 (0.2)	0.405
Wound disruption	14 (0.1)	5 (0.1)	0.644
Infectious complications, *n* (%)			
Pneumonia	61 (0.3)	26 (0.3)	0.787
Deep SSI	78 (0.4)	33 (0.3)	0.734
Sepsis	22 (0.1)	12 (0.1)	0.608
Medical complications, *n* (%)			
Unplanned intubation	40 (0.2)	22 (0.2)	0.469
Venous thromboembolism	95 (0.4)	52 (0.5)	0.277
Acute renal failure	27 (0.1)	15 (0.2)	0.530
Cardiac events	40 (0.2)	25 (0.3)	0.208
Cerebrovascular accidents	2 (0.01)	2 (0.02)	0.418
Composite outcomes, *n* (%)			
Serious complications	690 (3.1)	344 (3.4)	0.149
Mortality	48 (0.2)	29 (0.3)	0.222

*BS* bariatric surgery, *RYGB* Roux-en-Y gastric bypass, *SSI* surgical site infection

The overall 30-day postoperative complication rates were low and comparable between laparoscopic and robotic bariatric surgery groups. Surgical complications such as anastomotic or staple line leaks occurred in 0.3% of L-BS and 0.2% of R-BS patients (p = 0.214), while postoperative bleeding rates were 1.0% and 1.1%, respectively (p = 0.438). Reoperation rates (0.8% vs. 0.9%, p = 0.818), non-operative interventions (0.7% vs. 0.8%, p = 0.581), and readmissions (3.3% vs. 3.7%, p = 0.090) were similarly low. Medical complications, including cardiac events (0.2% vs. 0.3%, p = 0.208), pneumonia (0.3% both groups, p = 0.787), venous thromboembolism (0.4% vs. 0.5%, p = 0.277), and acute renal failure (0.1% vs. 0.2%, p = 0.530), did not differ significantly. Infectious complications such as deep surgical site infection (0.4% vs. 0.3%, p = 0.734) and sepsis (0.1% both groups, p = 0.608) were rare. The composite serious complication rate was 3.1% in the L-BS group and 3.4% in the R-BS group (p = 0.149), while 30-day mortality remained low at 0.2% for L-BS and 0.3% for R-BS (p = 0.222). The absolute risk difference for serious complications was 0.3% (95% CI: −0.1% to 0.7%), and for mortality was 0.1% (95% CI: −0.05% to 0.2%).

### Multivariable logistic regression for predictors of serious complications

Multivariable analysis identified several independent predictors of serious complications (Table 4): older age (OR 1.11 per 10 years, 95%CI 1.04–1.18, p = 0.002), hypertension (OR 1.60, 95%CI 1.38–1.86, p < 0.001), gastroesophageal reflux disease (OR 1.26, 95%CI 1.1–1.44, p = 0.001), history of myocardial infarction (OR 1.85, 95%CI 1.2–2.86, p = 0.005), therapeutic anticoagulation use (OR 1.77, 95%CI 1.43–2.18, p < 0.001), longer operative time (OR 1.11 per 30 min, 95%CI 1.07–1.15, p < 0.001), and RYGB (OR 1.55, 95%CI 1.34–1.78, p < 0.001). The robotic approach was not associated with an increased or decreased risk of serious complications (OR 0.99, 95% CI 0.87–1.14; p = 0.940). (AUROC = 0.65; Brier score = 0.03).

**Table 4:** Predict risk factors for serious complications

	Adjusted odds ratio^*^	95% Confidence interval	p-value
Higher BMI (per 5 kg/m^2^)	1.01	1.00—1.02	0.135
Older age (per 10 years)	1.11	1.04—1.18	0.002
Longer operative time (per 30 min)	1.11	1.07—1.15	< 0.001
Female	0.95	0.82—1.09	0.446
Diabetes			
Non-insulin dependent	0.95	0.81—1.11	0.507
Insulin dependent	1.08	0.86—1.36	0.504
Hypertension	1.60	1.38—1.86	< 0.001
GERD	1.26	1.10—1.44	0.001
Hyperlipidemia	1.06	0.90—1.25	0.493
History of myocardial infarction	1.85	1.20—2.86	0.005
Therapeutic anticoagulant	1.77	1.43—2.18	< 0.001
Robotic- vs Laparoscopic-assisted	0.99	0.87—1.14	0.940
RYGB vs SG	1.55	1.34—1.78	< 0.001

*BMI* Body mass index, *GERD* Gastroesophageal reflux disease, *RYGB* Roux-en-Y gastric bypass, *SG* Sleeve gastrectomy

## Discussion

In this large, national database analysis of bariatric surgery patients with BMI ≥ 60 kg/m2, we found no significant differences in 30-day serious complication or mortality rates between robotic and laparoscopic approaches. Despite a substantial increase in robotic utilization over the study period, perioperative safety profiles remained comparable, indicating that the potential technical advantages of the robotic platform have yet to demonstrate short-term clinical benefit in this high-risk cohort. Independent predictors of serious complications were driven primarily by patient comorbidities and procedural factors, rather than surgical approach, underscoring the importance of individualized risk stratification over platform selection in surgical planning for this population. However, these findings must be interpreted within the context of significant baseline differences between groups and potential unmeasured confounding factors that may have influenced surgical approach selection.

Interestingly, the rise in robotic utilization in our study mirrors the national pattern described by Bauerle et al. [7]. They reported more than a threefold increase in robotic use for both primary and revisional bariatric procedures from 2015 to 2020. Between 2020 and 2023, robotic utilization increased at an average rate of 7.3 percentage points per year. This rapid adoption of robotic technology in patients with BMI ≥ 60 kg/m2 occurred despite the absence of demonstrable short-term outcome advantages and was associated with a 27.5% increase in operative time. This change may reflect ergonomic benefits, enhanced visualization, and improved dexterity in constrained operative fields, though consistent short-term clinical advantages over laparoscopy remain unproven in large datasets across any surgical discipline [12, 16–18]. However, the absence of complication-related robotic superiority is particularly noteworthy given the increased healthcare costs burden associated with the robotic approach. The accelerated adoption pattern suggests that factors beyond patient outcomes, such as surgeon preference, marketing influences, institutional prestige, or perceived medicolegal advantages, may be driving technology adoption in this population.

Our findings of equivocal safety between approaches are also consistent with prior large-scale MBSAQIP analyses and systematic reviews looking at broad bariatric outcomes that have similarly demonstrated no significant differences in short-term morbidity, mortality, or overall complication rates between robotic and laparoscopic bariatric surgery. In a propensity-matched MBSAQIP cohort of 93,802 patients, Dudash et al. found no morbidity or mortality differences between robotic and laparoscopic SG or RYGB, despite longer robotic times (SG 89 vs 62 min; RYGB 141 vs 105 min [16]. Recent propensity-matched MBSAQIP analyses have reported largely comparable short-term outcomes between robotic and laparoscopic bariatric surgery, with heterogeneous, procedure-specific differences. Hope et al. found lower infection and bleeding rates for robotic RYGB but higher infection, bleeding, reoperation, and readmission rates for robotic sleeve gastrectomy, with longer operative times across procedures [19]. Similarly, Rahimi-Ardabily et al. demonstrated persistently longer operative times and higher healthcare utilization for robotic approaches without mortality differences [20]. However, the literature specific to patients with BMI ≥ 60 kg/m2 remains limited and the present study focuses exclusively on patients with BMI ≥ 60 kg/m2, a uniquely high-risk cohort in whom patient-level factors may attenuate detectable platform-specific differences. For patients with a BMI ≥ 50–60 kg/m2, several studies have shown that robotic bariatric surgery is as safe as laparoscopy, with no significant differences in mortality or overall complication rates, although for both RYGB and SG, robotics has been associated with fewer conversions and anastomotic leaks, but with longer operative times [21–23]. Additional evidence suggests potential technical advantages in selected subgroups: Gray et al. reported lower leak rates and fewer conversions in patients with super-super obesity [1]. Buchs et al. demonstrated zero conversion, and shorter hospital stays in complex revisional procedures, though with longer operative times (352 vs. 270 min) [24]. Importantly, most of these studies included mixed BMI populations or had limited power to detect differences in this high-risk group.

Although GERD itself may not directly cause postoperative complications, it likely that patients with GERD frequently have associated hiatal hernias, esophageal dysmotility, or a history of prior foregut surgery such as hiatal hernia repair, all of which may increase technical complexity and perioperative risk, particularly in patients with extreme obesity. In this context, GERD should be interpreted as a proxy for operative complexity and underlying disease burden rather than a direct causal factor. Taken together, these findings underscore that postoperative morbidity is likely driven more by the inherent complexity of the metabolic procedure and underlying patient factors than by the choice of surgical platform.

Proponents of laparoscopy may argue that without a complication advantage, robotics’ cost premium is notable: a meta-analysis of 14 studies (~ 1.41 M patients) found higher total (+ $3.8 k) and OR (+ $9.7 k) costs for robotic bariatric surgery, shorter operative times with laparoscopy, only trivial gains for robotics in length of stay and complications, and overall low-quality evidence [25], it calls into question the justification for its routine use, particularly given the increased costs and resource demands, which may be driven more by market forces, institutional prestige, or technology enthusiasm than by patient-centered outcome gains. Conversely, proponents of frequent robot use may argue that the absence of increased complications demonstrates acceptable safety in a new technology and may reduce the ergonomic strain associated with operating on patients with obesity, potentially lowering the risk of work-related musculoskeletal injuries and improving surgeon well-being when treating high-BMI populations [26]. However, Hider et al. reported that surgeons transitioning from stapled to hand-sewn anastomoses were more likely to adopt robotic surgery, which may reflect surgeon-specific practice patterns rather than inherent procedural advantages (27). The 27.5% increase in operative time associated with robotic surgery translates to approximately 23 additional minutes per case, which has implications for operating room efficiency, costs, and surgeon fatigue, particularly in high-volume centers. There is evidence to support both sides, however until further prospective trials provide justification for either modality, platform selection will continue to be guided by surgeon preferences, institutional resources, and ergonomic considerations rather than by patient outcomes.

Several limitations should be considered when interpreting these findings. First, there is a potential for unmeasured confounding exists despite adjustment for multiple patient- and procedure-level variables. The significant baseline differences observed between groups, including higher ASA classification, increased comorbidity burden, and demographic differences in the robotic cohort, suggest systematic differences in patient selection that may not be fully captured by available variables. Additionally certain variables, such as surgeon-level factors, including individual experience, technical preference, and institutional expertise with the robotic platform, are not reliably captured in MBSAQIP dataset, and may meaningfully impact outcomes. Additionally, the MBSAQIP dataset does not capture detailed foregut pathology, including the presence of hiatal hernia or prior foregut operations, which may increase technical complexity and perioperative risk. Finally, the thirty-day outcomes analyzed in the present study do not account for potential long-term benefits of robotics, which are valuable for comprehensive evaluation of this technology. Important outcomes such as incisional hernia rates, adhesion formation, chronic pain, or long-term surgeon ergonomic benefits are not captured in this analysis. Future prospective studies should incorporate granular operative metrics, surgeon experience levels, and long-term patient and surgeon-centered outcomes to more fully define the role of robotics in bariatric surgery in this population. Randomized controlled trials, while challenging to conduct, would provide the highest level of evidence for comparative effectiveness in SSO patients. Additionally, cost-effectiveness analyses incorporating both short- and long-term outcomes, as well as surgeon-reported outcomes related to ergonomics and fatigue, would inform evidence-based technology adoption decisions. Propensity score-matched analyses using institutional identifiers and surgeon-level variables could help address some of the selection bias limitations inherent in observational studies.

## Conclusion

In this national analysis of high-risk BMI ≥ 60 kg/m2 patients undergoing bariatric surgery, the robotic approach did not confer a short-term complication advantage over laparoscopy and was associated with 27% longer operative time. Despite significant baseline differences between groups suggesting potential selection bias, perioperative outcomes were primarily determined by patient comorbidities and procedural factors rather than surgical approach. While the robotic platform may offer technical and ergonomic benefits, these did not translate into demonstrable perioperative safety advantages in this cohort. The nearly threefold increase in robotic utilization over the study period occurred without corresponding outcome improvements, raising questions about the drivers of technology adoption. These findings suggest that neither approach demonstrates superior short-term safety in super-super obese patients, and that individualized risk stratification based on patient characteristics should guide perioperative planning rather than surgical platform selection. Future research should address selection bias through randomized trials and include cost-effectiveness analyses to better inform evidence-based technology adoption in this high-risk population.

## Supplementary Information

Below is the link to the electronic supplementary material.

Supplementary file1 (DOCX 146 kb)
