# Supplementary material for: Evaluating trends and outcomes between robotic and laparoscopic bariatric surgery in patients with BMI ≥ 60 kg/m2: an MBSAQIP analysis of 32,295 cases
Source: Surg Endosc. 2026 Mar 12;40(5):4309–16. doi: 10.1007/s00464-026-12652-5 (PMC13161256; doi:10.1007/s00464-026-12652-5)

**Supplement table legends**

- Table S1. Perioperative details and 30-day postoperative complications in RYGB subgroup.
- Table S2. Perioperative details and 30-day postoperative complications in SG subgroup.

| Table S1. Perioperative details and 30-day postoperative complications in RYGB subgroup. | | | |
| --- | --- | --- | --- |
|  | **L-RYGB** **(n = 5,872)** | **R-RYGB**  **(n = 2,767)** | **p-value** |
| **Operative year** |  |  | <0.0001 |
| 2020 | 1,491 (25.3) | 333 (12.0) |  |
| 2021 | 1,554 (26.5) | 570 (20.6) |  |
| 2022 | 1,483 (25.3) | 861 (31.1) |  |
| 2023 | 1,344 (22.9) | 1,003 (36.2) |  |
| **Operative details** |  |  |  |
| Operative time (mins), mean ± SD | 118.7 ± 55.0 | 146.5 ± 58.9 | <0.0001 |
| Length of stay (days), mean ± SD | 1.7 ± 1.8 | 1.5 ± 1.3 | <0.0001 |
| **Surgical complications, n (%)** |  |  |  |
| Readmission | 263 (4.5) | 153 (5.5) | 0.033 |
| Reoperation | 74 (1.3) | 37 (1.3) | 0.767 |
| Non-operative intervention | 76 (1.3) | 31 (1.1) | 0.495 |
| Leaks | 22 (0.4) | 9 (0.3) | 0.720 |
| Postoperative bleeding | 92 (1.6) | 49 (1.8) | 0.485 |
| Bowel obstruction | 26 (0.4) | 15 (0.5) | 0.531 |
| Wound Disruption | 7 (0.1) | 2 (0.1) | 0.528 |
| **Infectious complications, n (%)** |  |  |  |
| Pneumonia | 24 (0.4) | 13 (0.5) | 0.685 |
| Deep SSI | 32 (0.5) | 11 (0.4) | 0.364 |
| Sepsis | 9 (0.2) | 5 (0.2) | 0.767 |
| **Medical complications, n (%)** |  |  |  |
| Unplanned Intubation | 12 (0.2) | 6 (0.2) | 0.906 |
| Venous Thromboembolism | 29 (0.5) | 12 (0.4) | 0.704 |
| Acute renal failure | 10 (0.2) | 6 (0.2) | 0.639 |
| Cardiac events | 14 (0.2) | 8 (0.3) | 0.663 |
| Cerebrovascular accidents | 1 (0.02) | 0 (0) | 0.492 |
| **Composite outcomes, n (%)** |  |  |  |
| Serious Complications | 286 (4.9) | 131 (4.7) | 0.783 |
| Mortality | 16 (0.3) | 12 (0.4) | 0.219 |
| Abbreviations: L-, Laparoscopic; R-, Robotic; RYGB, Roux-en-Y gastric bypass; SSI, Surgical site infection | | | |

| Table S2. Perioperative details and 30-day postoperative complications in SG subgroup. | | | |
| --- | --- | --- | --- |
|  | **L-SG** **(n = 16,339)** | **R-SG**  **(n = 7,317)** | **p-value** |
| **Operative year** |  |  | <0.0001 |
| 2020 | 3,793 (23.2) | 1,025 (14.0) |  |
| 2021 | 4,448 (27.2) | 1,548 (21.2) |  |
| 2022 | 4,380 (26.8) | 2,184 (29.8) |  |
| 2023 | 3,718 (22.8) | 2,560 (35.0) |  |
| **Operative details** |  |  |  |
| Operative time (mins), mean ± SD | 70.9 ± 36.1 | 91.4 ± 38.7 | <0.0001 |
| Length of stay (days), mean ± SD | 1.4 ± 1.4 | 1.4 ± 1.4 | 0.639 |
| **Surgical complications, n (%)** |  |  |  |
| Readmission | 476 (2.9) | 220 (3.0) | 0.694 |
| Reoperation | 112 (0.7) | 50 (0.7) | 0.985 |
| Non-operative intervention | 79 (0.5) | 45 (0.6) | 0.195 |
| Leaks | 36 (0.2) | 10 (0.1) | 0.177 |
| Postoperative bleeding | 121 (0.7) | 57 (0.8) | 0.752 |
| Bowel obstruction | 5 (0.03) | 3 (0.04) | 0.688 |
| Wound Disruption | 7 (0.04) | 3 (0.04) | 0.949 |
| **Infectious complications, n (%)** |  |  |  |
| Pneumonia | 37 (0.2) | 13 (0.2) | 0.450 |
| Deep SSI | 46 (0.3) | 22 (0.3) | 0.799 |
| Sepsis | 13 (0.1) | 7 (0.1) | 0.694 |
| **Medical complications, n (%)** |  |  |  |
| Unplanned Intubation | 28 (0.2) | 16 (0.2) | 0.435 |
| Venous Thromboembolism | 66 (0.4) | 40 (0.6) | 0.129 |
| Acute renal failure | 17 (0.1) | 9 (0.1) | 0.684 |
| Cardiac events | 26 (0.2) | 17 (0.2) | 0.222 |
| Cerebrovascular accidents | 1 (0.01) | 2 (0.03) | 0.180 |
| **Composite outcomes, n (%)** |  |  |  |
| Serious Complications | 404 (2.5) | 213 (2.9) | 0.051 |
| Mortality | 32 (0.2) | 17 (0.2) | 0.568 |
| Abbreviations: L-, Laparoscopic; R-, Robotic; SG, Sleeve gastrectomy; SSI, Surgical site infection | | | |

ss

**Supplement figure legends**

- Figure S1. Temporal Trends in Mean Operative Time for Robotic and Laparoscopic Bariatric Procedures (2020–2023). (R-, Robotic; L-, Laparoscopic; RYGB, Roux-en-Y gastric bypass; SG, Sleeve gastrectomy) Created in https://BioRender.com


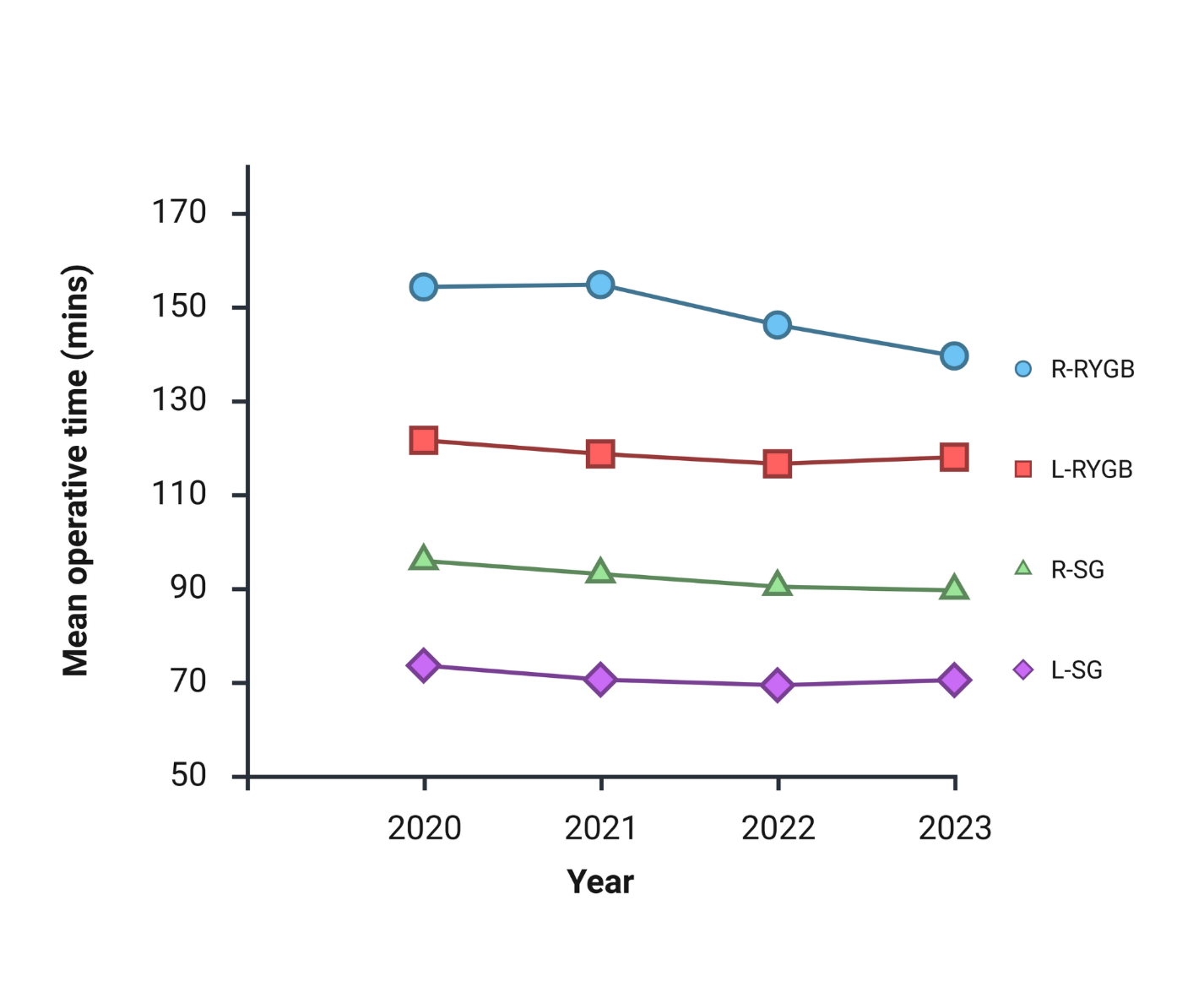

Supplement: Supplementary file 1 — Supplementary file1 (DOCX 146 kb) [file 464_2026_12652_MOESM1_ESM.docx]
